# Characterization of a GH5 endoxylanase from *Penicillium funiculosum* and its synergism with GH16 endo-1,3(4)-glucanase in saccharification of sugarcane bagasse

**DOI:** 10.1038/s41598-022-21529-1

**Published:** 2022-10-14

**Authors:** Olusola A. Ogunyewo, Omoaruemike E. Okereke, Sandeep Kumar, Syed Shams Yazdani

**Affiliations:** 1 Microbial Engineering Group, International Centre for Genetic Engineering and Biotechnology, New Delhi, 110067 India; 2 Systems Biology for Biofuel Group, International Centre for Genetic Engineering and Biotechnology, New Delhi, 110067 India; 3 DBT-ICGEB Centre for Advanced Bioenergy Research, International Centre for Genetic Engineering and Biotechnology, New Delhi, 110067 India; 4 Biotechnology Advanced Research Centre, Sheda Science and Technology Complex (SHESTCO), Abuja, Nigeria

**Keywords:** Biochemistry, Biotechnology, Microbiology, Molecular biology

## Abstract

The production of second-generation fuels from lignocellulosic residues such as sugarcane bagasse (SCB) requires the synergistic interaction of key cellulose-degrading enzymes and accessory proteins for their complete deconstruction to useful monomeric sugars. Here, we recombinantly expressed and characterized unknown GH5 xylanase from *P. funiculosum* (*Pf*Xyn5) in *Pichia pastoris*, which was earlier found in our study to be highly implicated in SCB saccharification. The *Pf*Xyn5 has a molecular mass of ~ 55 kDa and showed broad activity against a range of substrates like xylan, xyloglucan, laminarin and p-nitrophenyl-β-d-xylopyranoside, with the highest specific activity of 0.7 U/mg against xylan at pH 4.5 and 50 °C. Analysis of the degradation products of xylan and SCB by *Pf*Xyn5 showed significant production of xylooligosaccharides (XOS) with a degree of polymerization (DP) ranging from two (DP_2_) to six (DP_6_), thus, suggesting that the *Pf*Xyn5 is an endo-acting enzyme. The enzyme synergistically improved the saccharification of SCB when combined with the crude cellulase cocktail of *P. funiculosum* with a degree of synergism up to 1.32. The *Pf*Xyn5 was further expressed individually and simultaneously with a notable GH16 endoglucanase (*Pf*Egl16) in a catabolite-derepressed strain of *P. funiculosum*, *Pf*Mig1^88^, and the saccharification efficiency of the secretomes from the resulting transformants were investigated on SCB. The secretome of *Pf*Mig1^88^ overexpressing Xyn5 or Egl16 increased the saccharification of SCB by 9% or 7%, respectively, over the secretome of *Pf*Mig1^88^, while the secretome of dual transformant increased SCB saccharification by ~ 15% at the same minimal protein concentration.

## Introduction

The rapid increase in energy demand along with the continuous increase in the price of fossil fuels constantly necessitates the need to diversify the channels of energy generation for every growing economy^1–3^. The increasing shortage of non-renewable energy resources and huge environmental pressure from greenhouse gases released by the burning of fossil fuels continue to prompt growing studies in search of alternative production of cleaner fuels compared to petroleum-based fuels. Second-generation biofuels produced from lignocellulosic biomass continue to attract wide attention as bountiful sources as they neither compete with food requirements nor impact the food and feed markets^4,5^. Sugarcane bagasse (SCB) is one of the most abundant and promising biomass sources in the world which is obtained from the processing of sugarcane with a worldwide annual production of approximately 54 million tons^6^. On average, approximately 270 kg of bagasse (50% moisture) per metric ton of sugarcane is generated by the sugar factories as a major by-product^7^. SCB predominantly consists of cellulose, hemicellulose and lignin that are strongly associated to form the plant cell wall^8,9^.

The bioconversion process of this biomass requires several arrays of lignocellulolytic enzymes which act synergistically to deconstruct the tightly packed polymeric carbohydrate components into monomeric sugars that can subsequently be converted to fuels and valuable chemicals. Previous studies have shown that several biomass conversion processes rely on biomass pretreatment before enzymatic hydrolysis, to remove lignin and reduce the recalcitrance nature of the biopolymer^10–12^. However, this process often leads to a reduction in the hemicellulose content^13^. Owing to this challenge, several mild pretreatment techniques are getting developed to reduce the loss in hemicellulose content of lignocellulosic biomass thereby giving attention to more characterization of hemicellulases and notable accessory enzymes beneficial to biomass deconstruction^10^. Hemicellulases such as xylanases, lichenases and laminarases which are endoglucanases active on mixed-glucans have earlier been reported to improve the hydrolysis of xylan and cellulose thereby contributing to the reduction of enzyme dosage for complete biomass saccharification^13,14^. Xylanases (EC. 3.2.1.8), are crucial enzymes employed for the hydrolysis of β-1,4-d-xylosidic linkages in the xylan biomass, releasing xylooligosaccharides (XOS) and xylose. This enzyme has been reported to demonstrate wide application in several industrial processes such as in biorefinery, food and biobleaching of paper and pulp. It has been shown that xylanase treatment reduces the content of hexenuronic acid (HexA) in the pulp. HexA is a component formed during chemical pulping from glucuronic acid, which is part of hemicellulose polymers^15^. Based on the sequence and protein structure, xylanases are classified into many glycosyl hydrolase families (GH) such as 7, 8, 10, 11, 16, 30, 43, 51, 98, and 141, with most of the characterized fungal xylanases being GH 10 and 11^16^. While there are extensive studies on GH5 xylanases from bacterial hosts, there is a dearth of information on fungal GH5 xylanase till date. Furthermore, due to the presence of β-(1,3)- and β-(1,4)-d-glucans in plant cell walls, the requirements for mixed-linked glucanases such as endo-1,4-β-glucanase (EC 3.2.1.4), endo-1,3(4)-β-glucanase (EC 3.2.1.6), endo-1,3-β-glucanase (EC 3.2.1.39), and endo-1,3–1,4-β-glucanase (EC 3.2.1.73) in cellulolytic enzyme cocktails could also be important for better saccharification performance^17^.

In an attempt to develop effective technology for utilization of SCB in biorefineries, especially for production of second-generation biofuels, we previously identified the fungus *Penicillium funiculosum* NCIM1228 as a suitable host for the production of potent lignocellulolytic enzymes for the deconstruction of diverse pretreated cellulosic feedstocks as its secretome exhibited outstanding saccharification potential when compared with secretomes from other fungal strains^18,19^. Using a combination of both proteomic and biochemical approaches, we identified some relevant accessory enzymes such as xylanases (GH30), endo‑β‑1,3(4)‑glucanase (GH16), β‑1,3‑galactosidase (GH43), cutinase (CE5), and some glycoside hydrolases with unknown functions important for the conversion of SCB to monomeric sugars when the production media for cultivation of *Penicillium funiculosum* was modulated with SCB^14^. It was seen that the remarkable induction of these enzymes in the secretome of *P. funiculosum* facilitated complete saccharification of SCB although at a high enzyme dosage.

Therefore, this study was designed to further improve the quality of enzyme systems in the secretome of *P. funiculosum* for enhanced saccharification of SCB at low enzyme loading to reduce the overall enzyme requirement for SCB deconstruction. To achieve this, two accessory enzymes, i.e., a GH5 protein with an unknown function and a GH16 endoglucanase, both found to be highly upregulated in the secretome of *P. funiculosum* in the presence of SCB, were selected for overexpression in *P. funiculosum*. The unknown function enzyme from *P. funiculosum* belonging to glycosyl hydrolase family 5 (*Pf*GH5) was first recombinantly expressed in *Pichia pastoris* and its characteristics were evaluated to decipher its role in biomass hydrolysis. Furthermore, its impact on SCB hydrolysis was investigated and then it was overexpressed along with a GH16 endoglucanase in the background of a catabolite-derepressed strain of *P. funiculosum* (*Pf*Mig1^88^) to produce a more efficient cocktail specific for this biomass. The resulting enzyme cocktail from the engineered strain was then assessed for improved production of hemicellulolytic enzymes as well as for their saccharification efficiency toward SCB with minimal enzyme loading.

## Results and discussions

### Sequence analysis of an uncharacterized GH5 family protein of *P. funiculosum*

**Figure 1:**
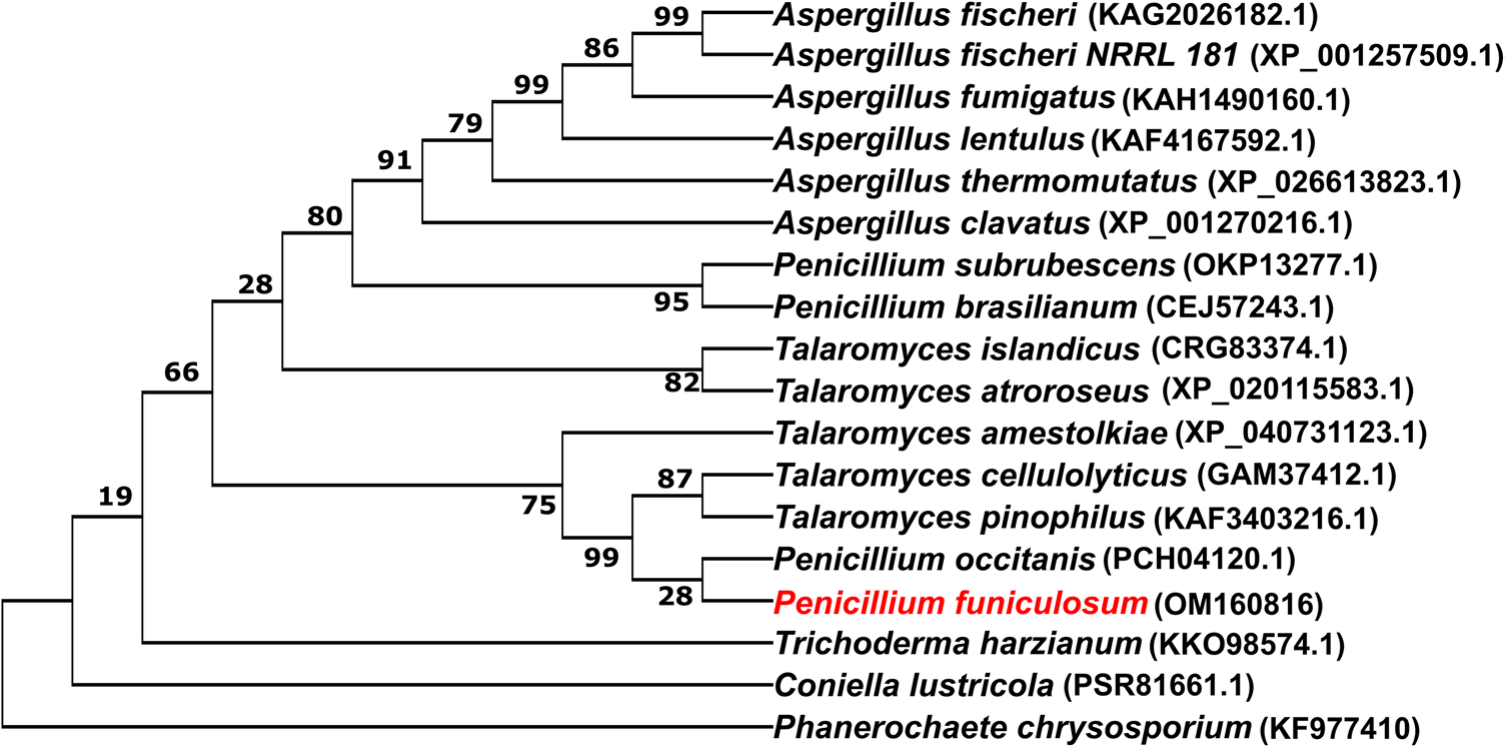
Phylogenetic tree of *Pf*Xyn5 orthologs in fungi. Molecular phylogenetic analysis was performed using the maximum likelihood method and the JTT matrix-based model. The tree with the highest log likelihood (− 17,992.93) is shown. Initial trees for the heuristic search were obtained automatically by applying neighbor-joining and BioNJ algorithms to a matrix of pairwise distances estimated using the maximum composite likelihood (MCL) approach and then selecting the topology with a superior log likelihood value. The bootstrap support corresponding to the numbers on the tree branches was calculated per 1000 bootstrap replicates^39^. This analysis involved the catalytic domains of 18 closely related proteins. Evolutionary analyses were conducted using MEGA X software.

A unique GH5 family protein was one of the proteins that was found to be highly upregulated in our earlier study in the secretome of *P. funiculosum* in response to the pre-treated sugarcane bagasse and was implicated in the enhanced hydrolysis of the pretreated bagasse by the secretome^14^. The sequence analysis of this GH5 family protein of *P. funiculosum*, *Pf*GH5, showed that this protein has not yet been characterized since the functions of the most similar genes that have been deposited in NCBI as well as in the CAZY database are unknown. As a result, we attempted to characterize and assess the functionality of this gene. The sequence analysis indicated that the *Pf*GH5 contains 1561 nucleotides which encode a chain of 454 amino acid residues with a theoretical molecular weight of 50.1 kDa. Further analysis showed that there were four potential N-glycosylation sites (Asn-Ala-Ser) found in the *Pf*GH5 sequence using the NetNGlyc1.0 server (http://www.cbs.dtu.dk/services/NetNGlyc/), while there was no potential O-glycosylation predicted using the NetOGlyc 4.0 server (http://www.cbs.dtu.dk/services/NetOGlyc/). The *Pf*GH5 amino acid sequence revealed 100% similarity to an uncharacterized glycoside hydrolase family 5 protein from *P. occitanis* (accession number PCH04120.1), while 99% similarity was found with *Talaromyces cellulolyticus* (GAM37412.1) and *Talaromyces pinophilus* (KAF3403216.1) as seen also from the phylogenetic tree (Fig. 1). Surprisingly, the protein also exhibited 82% similarity with a *Talaromyces islandicus* GH5 protein annotated to be a β-xylosidase suggesting its possibility as a xylan-degrading enzyme. Further analysis using the InterPro scan tool revealed the presence of a conserved domain of cellulase super-family glycoside hydrolase 5 between positions 162–171. The nucleotide sequence of *Pf*GH5 was deposited in the NCBI database with accession number OM160816.

### Recombinant expression, purification and functional characteristics of *Pf*GH5 protein

**Figure 2:**
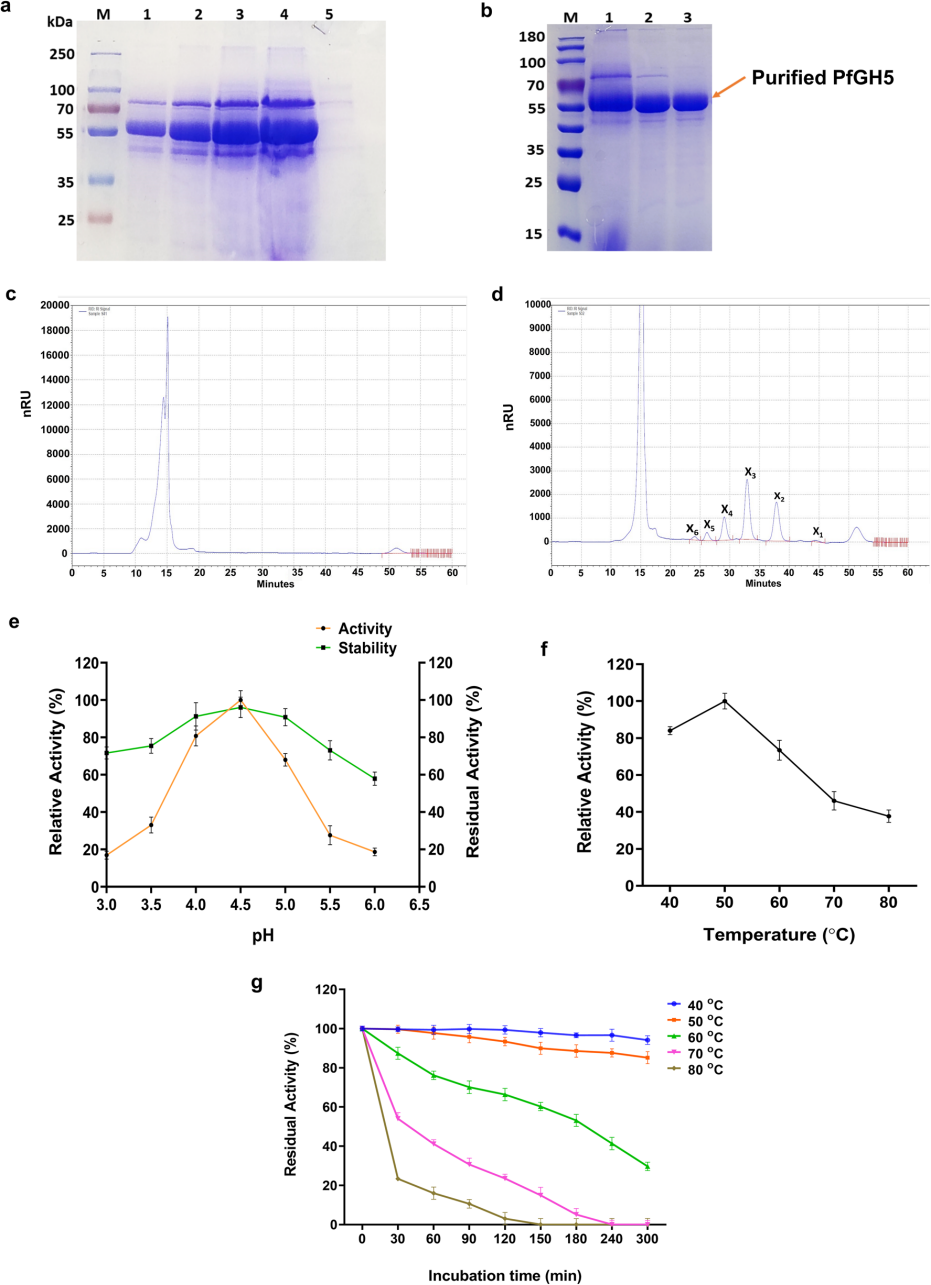
Expression and characterization of recombinant *Pf*Xyn5 in *P. pastoris* (**a**) SDS-PAGE gel of the supernatant of PfXyn5 produced by *P. pastoris* over 96 h period of induction. Lanes 1–4 refers to induced cultures of PfXyn5 taken at 24, 48, 72 and 96 h time point while lane 5 refers to wild type culture of *P. pastoris* X-33 at 96 h time point. (**b**) SDS-PAGE gel of purified fractions of *Pf*Xyn5 by anion exchange chromatography using Q-Sepharose Fast Flow resin. Lane 1 refers to the crude enzyme while lanes 2 and 3 refer to fractions eluted with 25 and 50 mM NaCl gradient. (**c**) HPLC profile of hydrolysed products of beechwood xylan without *Pf*Xyn5 (control). (**d**) HPLC profile of hydrolysed products of beechwood xylan with *Pf*Xyn5. X_1_, X_2_, X_3_, X_4_, X_5_ and X_6_ refers to xylose, xylobiose, xylotriose, xylotetrose, xylopentaose and xylohexaose, respectively. (**e**) Effects of pH on *Pf*Xyn5 activity and stability. The relative activity (%) was calculated relative to the optimum activity obtained at pH 4.5 which was taken as 100%. (**f**) Effects of temperature on *Pf*Xyn5 activity. The relative activity (%) was calculated relative to the optimum activity obtained at 50 °C which was taken as 100% (**g**) Thermostability profile of *Pf*Xyn5. Stability at 40 °C, 50 °C, 60 °C, 70 °C and 80 °C were determined by incubating enzyme at specified temperatures for 300 min while samples were withdrawn every 30 min before assaying for residual activity according to the standard assay procedure.

To understand the properties of this *Pf*GH5 protein and its role in biomass deconstruction, the protein was first recombinantly expressed using *Pichia pastoris* as a host. *P. pastoris* holds a great advantage as a system for the production of recombinant proteins because of its ability to produce highly efficient enzymes in high abundance as well as for fast growth characteristics in inexpensive media^20^. In addition, its capability for appropriate folding and transportation of proteins renders it an excellent tool for recombinant protein production^20^. *P. pastoris* also secretes fewer native proteins thereby enabling easier purification and biochemical characterization of recombinant proteins in supernatant^21,22^. To express this protein, the alpha secretion signal peptide of the *p*PICZαA vector was used to direct the secretion of the protein under the AOX1 promoter. For this, the *Pf*GH5 was fused with the α-secretion signal of the *p*PICZα vector to obtain the vector *p*PICZαA-*Pf*GH5, designated as *p*OAO6. The recombinant *p*OAO6 vector was linearized with *Sac*I restriction enzyme to enable its integration into the *P. pastoris* X-33 genome by homologous recombination. Linearized vector was then transformed into competent *P. pastoris* X-33 for expression and the transformants were selected with Zeocin selection marker (100 μg/ml) for 72 h. Some positive transformants were randomly picked up and re-cultivated on YPDA plates at 30 °C with different zeocin concentrations between 200 and 1000 μg/ml for screening multi-copy positive transformants. A strain with multi-copy integration of the gene that resisted 1000 μg/ml zeocin was further selected, confirmed by PCR (Supplementary Fig. S1) and then inoculated into 50 ml inducing media at 30 °C and 220 rpm for producing the recombinant PfGH5. The induction was monitored every 24 h by loading some of the culture supernatants on SDS-PAGE electrophoresis (Fig. 2a; Supplementary Fig. S2a) and the recombinant enzyme was finally recovered from *Pichia pastoris* culture supernatant at approximately 1.8 g/L protein after 96 h of induction.

*Pf*GH5 was purified to apparent homogeneity by a single step of ion-exchange chromatography using Q-Sepharose Fast Flow (QSFF) and the purified enzyme was then used for all subsequent biochemical characterization (Supplementary Fig. S2b). The purified enzyme showed an intense single band on SDS-PAGE with a molecular mass close to 55 kDa (Fig. 2b), which was slightly higher than the theoretical mass of 50 kDa. The difference in sizes suggested that post-translational modifications occurred in *Pf*GH5 during heterologous expression in *P. pastoris* and these post-translational modifications had effects on the migration rate of the *Pf*GH5 on SDS-PAGE^23^. To gain insights into the action of this glycoside hydrolase in biomass deconstruction, the enzyme was screened on various polysaccharides, for its specificity on glycosidic bonds (Table 1). The *Pf*GH5 exhibited the highest activity toward beechwood xylan with a specific activity of 0.7 U/mg protein followed by xyloglucan and lichenan. The results also showed that the *Pf*GH5 could also hydrolyse laminarin, carboxymethyl cellulose and para-nitrophenyl-β-d-xylopyranoside (pNPX) although the efficiencies were much lower than with xylan while there was no activity detected on Avicel, β-glucan and para-nitrophenyl-β-d-glucopyranoside (pNPG) (Table 1).

**Table 1:** Substrate specificity of the recombinant *Pf*GH5 on various polysaccharides.

Substrate	Avicel	CMC	Xylan	Lichenan	Laminarin	Xylo-glucan	β-glucan	pNPX	pNPG
Specific activity (U/mg) of *Pf*GH5	ND	0.011 ± 0.01	0.792 ± 0.11	0.113 ± 0.01	0.033 ± 0.01	0.240 ± 0.05	ND	0.009 ± 0.001	ND

*ND* no detected activity.

Recognition of a broad range of substrates for catalysis indicated the promiscuous nature of the enzyme. However, the relatively higher specific activity of this enzyme towards both xylan and xyloglucan when compared with other substrates screened suggests the possibility of this *Pf*GH5 be mainly a xylanase. Therefore, to further understand the mechanism of action of the enzyme and the nature of the products it produces, the hydrolysis reactions were set up with 5% xylan as well as with 5% pretreated SCB as the substrate and 25 mg/g protein dosage at 50 °C for 24 h and 72 h, respectively. Analysis of the hydrolysate showed significant production of xylooligosaccharides (XOS) with a degree of polymerization (DP) up to six (DP_6_) from the xylan in the test reaction containing the enzyme, while there was no trace of XOS in control reactions without enzyme in the reaction mixture after 24 h of incubation (Fig. 2c,d). We observed significant liberation of xylobiose and xylotriose with a very less amount of xylose in the hydrolysate. This mode of action was found to be similar to that of an endoxylanase from *Trichoderma asperellum*^24^, thus, suggesting that the *Pf*GH5 is an endo-acting enzyme that randomly cleaves the β-linkages in xylan^25^. Furthermore, the chromatogram of the hydrolysate for the SCB test condition showed the presence of some glucose released in addition to xylose and XOS by *Pf*GH5 (Supplementary Fig. S3a,b). The detected glucose in the SCB hydrolysates could be from the breakdown of xyloglucan or other mixed-linked glucans that are an integral component of the hemicellulose complex in SCB, as the enzyme showed some activities towards pure xyloglucan as well as mixed-linked glucan substrates (Table 1).

To further characterize the enzyme, the effect of pH on the *Pf*GH5 xylanase activity was examined from pH 3.0 to 6.0. The results showed that the enzyme exhibited maximum activity at pH 4.5, while it had a relative activity of 76% and 60% at pH 4.0 and 5.0, respectively (Fig. 2e). The optimum pH activity observed with this enzyme is in contrast to earlier characterised GH5 xylanases mostly of bacterial origin which exhibited optimum activity between pH 5 and 6^26,27^. The enzyme exhibited good stability at room temperature between pH 3.5–5.5 after 24 h of incubation (Fig. 2e). It was most stable at pH 4.5 and retained about 96% of its original activity after 24 h of incubation at this pH. It was remarkable that the enzyme had a residual activity of 90% at both pH 4.0 and 5.0 after 24 h of incubation (Fig. 2e). The high activity of this enzyme at acidic pH range and stability across a wider pH range indicates that this enzyme could be potentially relevant as most saccharification assays with fungal enzymes are mostly performed at acidic conditions between pH 4 and 5^28^. Evaluation of the temperature optima of this enzyme showed that *Pf*GH5 displayed maximal activity at 50 °C while it exhibited 84% and 73% of its activity at 40 °C and 60 °C, respectively (Fig. 2f). The thermostability of the enzyme was monitored by incubating enzyme at various elevated temperatures for 5 h and then measuring its residual activity. The enzyme was found most stable between 40 and 50 °C where it retained 85% of its original activity at 50 °C after 5 h of incubation (Fig. 2g). At more elevated temperatures (70 and 80 °C), the activity declined drastically (Fig. 2g). The half-lives of *Pf*GH5 at 60, 70 and 80 °C were 192, 45 and 10 min, respectively (Table 2; Supplementary Fig. S4). Significant enzyme activity at elevated temperature is advantageous for industrial biomass processing, as it not only improves substrate solubility and decreases viscosity but also minimizes microbial contaminations^29^.

**Table 2:** Half-life of *Pf*GH5 after incubation at different temperatures.

Temp.	40 °C	50 °C	60 °C	70 °C	80 °C
T_1/2_ (min)	5293	1885	192	45	10

### Synergistic performance of *Pf*GH5 with cellulases in saccharification of SCB

Since most of the characterized xylanases from the GH10 and GH11 families have been reported as notable accessory enzymes which work synergistically with cellulases in biomass deconstruction^30,31^, we next evaluated the impact of this new enzyme in hydrolysis of complex heterogeneous biomass together with the secretome from *P. funiculosum* (*Pf*Mig1^88^). For this experiment, pretreated SCB was used as cellulosic substrate. The hydrolysis reaction was set-up at 5% solid loading and a total protein dosage of 10 mg/g dry biomass weight (DBW) that was modulated in different ratios by replacing a portion of the *P. funiculosum* cellulase cocktail with *Pf*GH5 xylanase for 72 h at 50 °C as shown in Table 3. For the enzyme replacement approach, varying amounts of the *Pf*Mig1^88^ cellulases were replaced with *Pf*GH5 xylanase, while the total amount of enzyme added was kept constant on a protein basis at 10 mg/g DBW. Interestingly, the supplementation of the cellulase cocktail with varying amounts of *Pf*GH5 xylanase increased the saccharification potential of *P. funiculosum* cellulase cocktail synergistically. The results showed substantial boosting effect in hydrolysis with the 80:20 and 90:10 enzyme combination ratio, while the 95:5 ratio showed maximum improvement and the highest degree of synergism (Table 3). The synergistic effect between this accessory enzyme and the cellulase cocktail could be due to the creation of more binding sites as a result of the removal of non-cellulosic polysaccharides by the xylanase for cellulase to act upon^32^. It was noticed that the total amount of monomeric sugars released with the 95:5 ratio increased by ~ 32% (10.78 g/L) as compared to the 100:0 ratio without the addition of *Pf*GH5 (8.15 g/L), thus indicating that this enzyme holds great biotechnological prospect in improving the saccharification performance of *P. funiculosum* at reduced cellulase loading.

**Table 3:** Degree of synergism of *P. funiculosum* with *Pf*GH5 xylanase in SCB saccharification.

Ratio of *P. funiculosum* secretome with PfGH5 xylanase	Glucose yield (g/L)	Xylose yield (g/L)	Arabinose yield (g/L)	Total sugar (g/L)	Degree of synergism
60:40	4.84 ± 0.14	2.91 ± 0.04	0.16 ± 0.00	7.91 ± 0.25	0.97
70:30	5.16 ± 0.06	3.16 ± 0.10	0.13 ± 0.02	8.45 ± 0.21	1.04
80:20	5.34 ± 0.29	3.79 ± 0.17	0.15 ± 0.01	9.23 ± 0.11	1.13
90:10	5.43 ± 0.28	4.03 ± 0.14	0.14 ± 0.01	9.60 ± 0.16	1.18
95:05	5.39 ± 0.27	5.23 ± 0.18	0.16 ± 0.02	10.78 ± 0.28	1.32
100:0	5.06 ± 0.18	2.95 ± 0.38	0.14 ± 0.01	8.15 ± 0.02	NA

A DS higher than one indicates there is cooperation among the involved enzymes while DS lower than one indicates there is competition between the involved enzymes.

*NA* not applicable.

### Vector construction and overexpression of ***Pf***Xyn5 in catabolite derepressed strain of ***Pf***Mig1^88^

**Figure 3:**
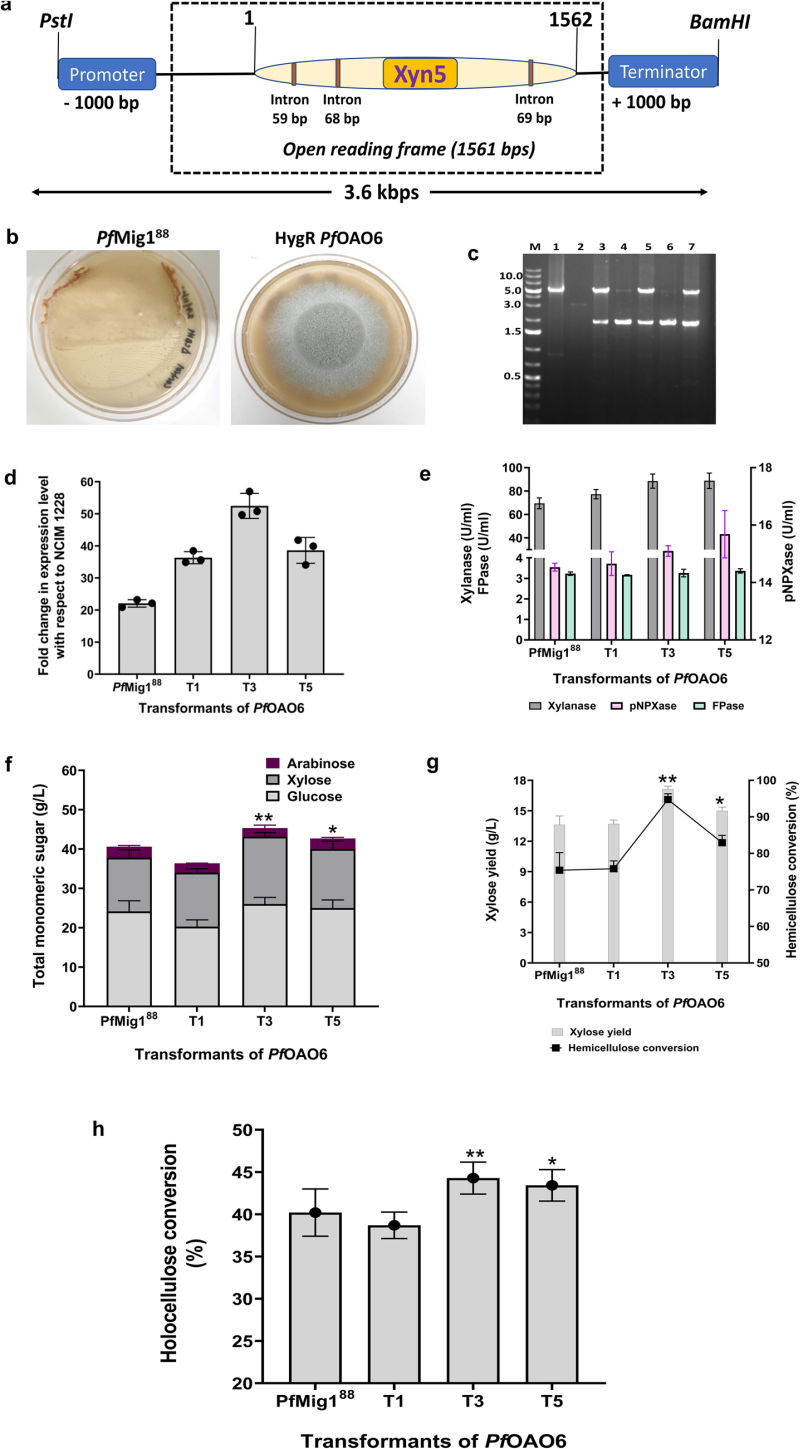
Construction of the *Pf*Xyn5 expression cassette and its overexpression in *Pf*Mig1^88^. (**a**) Schematic diagram showing the assembly of the *Pf*Xyn5 cassette from the *P. funiculosum* NCIM1228 genome. (**b**) Transformants of pOAO7 after AMTM transformation in *Pf*Mig1^88^. Transformants were selected on 100 µg/ml hygromycin. (**c**) Determination of *Pf*Xyn5 integration in the genome of *Pf*Mig1^88^ transformants by PCR using the primers PgpdA-F and TrpC-R. The expected size is 5 kbp, corresponding to a fragment spanning the region of gpdA promoter and TrpC on the expression cassette. Lane M is DNA molecular mass marker; lane (1) is the pOAO7 plasmid which served as a positive control while lane (2) is the gDNA of *Pf*Mig1^88^ which was the negative control. Lanes 3–7 are the transformants of P*f*OAO6. (**d**) The transcriptional expression of xylanase in NCIM1228, *Pf*Mig1^88^, and all the three positive strains of *Pf*OAO6 was measured by quantitative real-time PCR after growing the strains for 60 h in the presence of Avicel and SCB. The expression levels were normalized to those in NCIM1228 and plotted. (**e**) The xylanase, β-xylosidase and FPase activities in the fermentation broth of *Pf*Mig1^88^ and three transformants of *Pf*OAO6. (**f**) Total sugar released from hydrolysis of SCB by secretomes of *Pf*Mig1^88^ and three transformants of *Pf*OAO6. (**g**) Saccharification performance of *Pf*OAO6 secretomes on SCB. Total xylose yield was estimated after 72 h hydrolysis and the hemicellulose conversion were determined and plotted (**h**) Percentage holocellulose conversion measured at 72 h saccharification period. ** shows the significant difference at α = 0.05 using Tukey’s multiple comparison test.

Upon validation of the function of this GH5 protein to be a xylanase, it was further designated as *Pf*Xyn5. Also, from the promising boosting characteristics it exhibits in SCB saccharification when combined with cellulase cocktail, we next decided to overexpress this gene in the background of the Mig1-repressor-deleted strain of *P. funiculosum*, i.e., *Pf*Mig1^88^, as it has been shown to produce a larger amount of cellulolytic enzymes in our earlier study^33^. To overexpress the xylanase gene in *Pf*Mig1^88^, the expression vector containing the endogenous gene along with its promoter and terminator was constructed using the backbone of pBIF binary vector as mentioned in the method section. The 3.6-kb region containing the Xyn5 gene (Fig. 3a) was amplified from the genome of the parent NCIM1228 strain and cloned into the *p*BIF vector to generate the *p*OAO7 plasmid. The *p*OAO7 recombinant plasmid was confirmed by restriction digestion (Supplementary Fig. S5a) before transformation into the *Pf*Mig1^88^ strain using the agrobacterium-mediated transformation method (AMTM). The hygromycin-resistant transformants obtained (Fig. 3b) were subsequently analysed by performing PCR to check for the integration of the new expression cassette into the genome of the fungus (Fig. 3c). The amplification of the expected 5 kb fragment in three of the selected transformants (*Pf*OAO6) and found to be absent in the control (*Pf*Mig1^88^) confirmed the integration of the Xyn5 cassette into the genome of the fungus.

To understand the effect of increasing the copy number of this enzyme on its expression, the transcript abundance of the transformants was next examined at the mRNA level through quantitative PCR (qPCR). For this, cultures of NCIM1228, *Pf*Mig1^88^, and the three positive transformants of *Pf*OAO6 were grown in Mandel’s media containing 25 g/L Avicel and 15 g/L SCB for 60 h to obtain good mycelial growth. The transcript levels of all five strains under derepressing conditions were determined by real-time PCR (RT-PCR) with tubulin as a control, and the relative fold change was normalized to the level in NCIM1228 since it was the original strain from which all other mutant strains (*Pf*Mig1^88^ and *Pf*OAO6) were generated (Fig. 3d). The results under the overexpression showed about a 20-fold increase in Xyn5 transcripts for *Pf*Mig1^88^, while there were about 35 to 40-fold increases in transcript levels for the *Pf*OAO6 strains. To evaluate the expression level of the xylanase gene in the *Pf*OAO6 transformants, the three transformants with increased transcripts were cultivated in a cellulase-inducing medium (CIM) for 5 days. The transformants were then screened based on the xylanase production capability of their culture supernatant and the results were compared with those for the parent strain, *Pf*Mig1^88^ (Fig. 3e). The results showed a significant increase in xylanase activity in the range of 11 to 27%, in all the transformants compared to the parent strain, with the transformant T3 showing maximum xylanase production (Fig. 3e). Furthermore, we investigated if the overexpression of this gene will have an impact on the β-xylosidase content in the secretome since it is the last enzyme in the xylan degradation system that converts xylobiose to xylose. We found a close to ~ 9% increase in the β-xylosidase activity in two of the transformants (T3 and T5) while the β-xylosidase activity of transformants T1 was the same as the control strain (*Pf*Mig1^88^). It was observed that these differences could be due to the differences in the integration loci of the expression cassette in the *Pf*Mig1^88^ genome when performing random integration as seen in our earlier study^34^. As expected, we saw that there were no changes in the FPase activity of the secretomes in comparison with that of the parent strain (Fig. 3e), as the GH5 xylanase was found not to be active on a pure cellulosic substrate which is the main substrate in the standard filter paper assay reaction mixture (Table 1).

Since there was a significant improvement in the xylanase content of the transformants’ secretome as seen both from the transcriptional and translational studies, we next evaluated its corresponding effect on the digestion of pretreated SCB (Fig. 3f–h). For this, an SCB hydrolysis reaction was set up with a 15% solid load and minimal protein loading of 2.5 FPU/g DBW at 50 °C for 72 h. A low dose of 2.5 FPU/g was considered to avoid complete saccharification of the SCB, thereby enabling easy identification of the differences in the monomeric sugars released if any in the hydrolysate. The results obtained after hydrolysis showed no difference in the total glucose released from the secretomes of the transformants when compared with the native strain (Fig. 3f), while there was a noticeable improvement in the xylose content after 72 h as expected. The xylose yield with the secretomes of transformants T3 and T5 increased to 17 g/L which corresponded to 94% hemicellulose conversion when compared with the control that yielded 13.6 g/L xylose which corresponded to 76% hemicellulose conversion (Fig. 3g). Overall, the improvement in hemicellulose conversion in the transformants resulted in 44% total holocellulose conversion (Fig. 3h) which was ~ 9% improvement over that of the parent strain. The results indicate that the gene was successfully overexpressed and the integration of the additional copy of this gene in the fungus genome indeed helped in further improving the saccharification efficiency of its secretome in SCB deconstruction.

### Overexpression of a GH16 endo‑β‑1,3(4)‑glucanase (***Pf***Egl16) in ***Pf***Mig1^88^ significantly improved glucose yield from SCB

**Figure 4:**
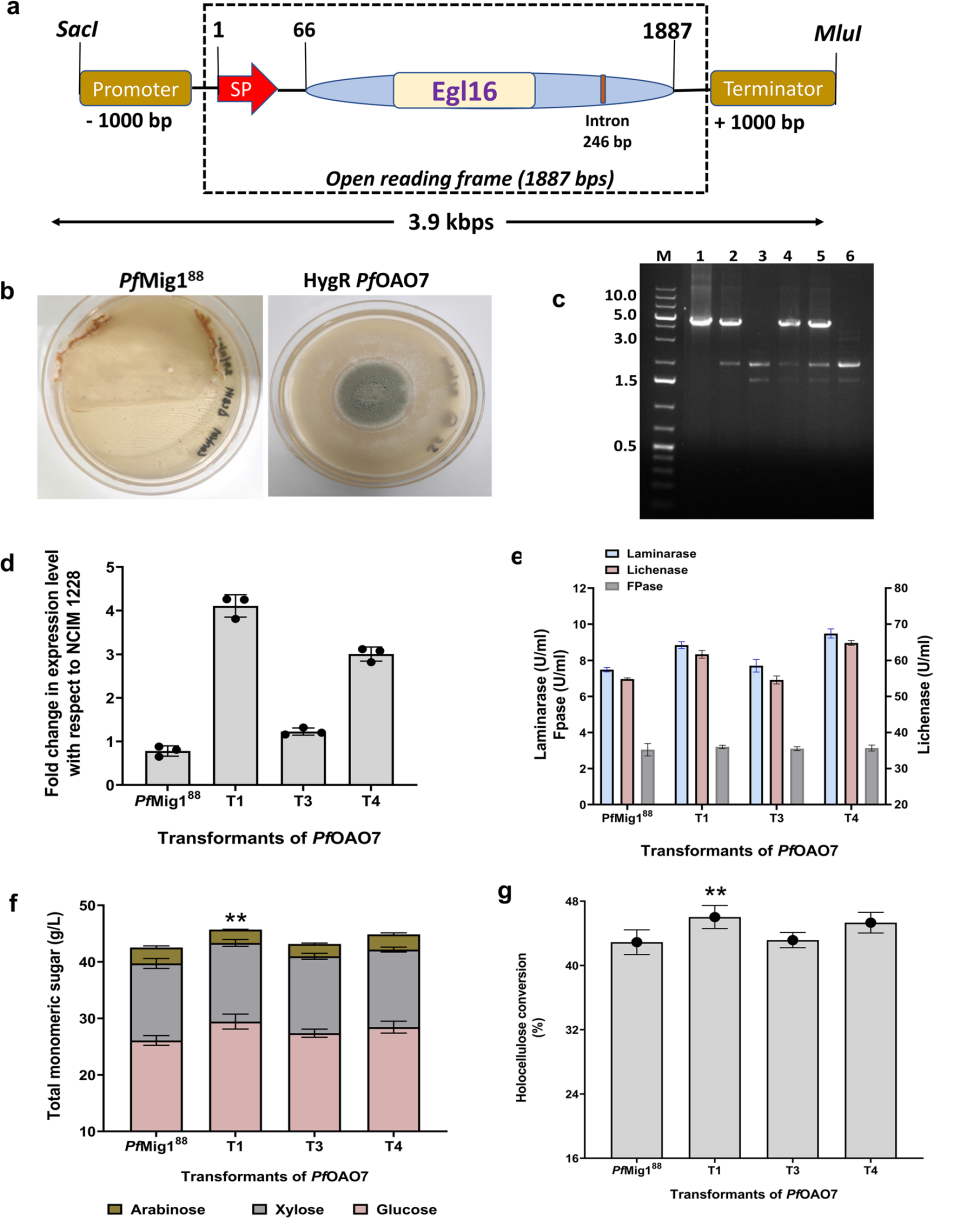
Construction of the *Pf*Egl16 expression cassette and its overexpression in *Pf*Mig1^88^. (**a**) Schematic diagram showing the assembly of the *Pf*Egl16 cassette from the *P. funiculosum* NCIM1228 genome. (**b**) Transformants of pOAO9 after AMTM transformation in *Pf*Mig1^88^. Transformants were selected on 100 µg/ml hygromycin. (**c**) Determination of *Pf*Egl16 integration in the genome of *Pf*Mig1^88^ transformants by PCR using the primers PgpdA-F and TrpC-R. The expected size is 5 kbp, corresponding to a fragment spanning the region of gpdA promoter and TrpC on the expression cassette. Lane M is DNA molecular mass marker; lane (1) is the pOAO9 plasmid which served as a positive control while lanes 2–5 are the transformants of *Pf*OAO7. Lane (6) is the gDNA of *Pf*Mig1^88^ which was the negative control. (**d**) The transcriptional expression of endoglucanase in NCIM1228, *Pf*Mig1^88^, and all the three positive strains of *Pf*OAO7 was measured by quantitative real-time PCR after growing the strains for 60 h in the presence of Avicel and SCB. The expression levels were normalized to those in NCIM1228 and plotted. (**e**) The lichenase, laminarase and FPase activities in the fermentation broth of *Pf*Mig1^88^ and three transformants of *Pf*OAO7. (**f**) Saccharification performance of *Pf*OAO7 secretomes on SCB. Total sugar released from hydrolysis of SCB by secretomes of *Pf*Mig1^88^ and three transformants of *Pf*OAO7. (**g**) Percentage holocellulose conversion measured at 72 h saccharification period. ** shows the significant difference at α = 0.05 using Tukey’s multiple comparison test.

Since the overexpression of the PfXyn5 gene in *Pf*Mig1^88^ from the earlier section improved only the saccharification of the hemicellulose component of SCB with almost no effect on the cellulose conversion, we next focused on overexpressing another gene, a GH16 endo‑β‑1,3(4)‑glucanase (Egl16) with accession number OM483862, which was also significantly upregulated in the presence of SCB from earlier proteomics study^14^. This enzyme is one of the β-glucanases typically active on complex polysaccharides containing mixed linkages such as β-(1,3)- and β-(1,4)-d-glucans in plant cell wall^35^. To overexpress the Egl16 gene of *P. funiculosum* in *Pf*Mig1^88^ strain, the Egl16 nucleotide which consists of 1887 bp with one intron and 546 amino acids was constructed using the backbone of the *p*BIF binary vector along with its native promoter and terminator (Fig. 4a). The gene was amplified from the genome of the parent strain and first cloned into a PCR cloning vector pJET1.2 to generate the *p*OAO8 plasmid. The *Pst*I/*Xba*I fragment was excised and cloned into the same sites in pBIF to create the *p*OAO9 vector. The *p*OAO9 plasmid was then digested with *Mun*I and *Mlu*I restriction enzymes to confirm the presence of the Egl16 gene in the expression cassette and absence in the pBIF vector (Supplementary Fig. S5b). The confirmed *p*OAO9 plasmid was then transformed into *Pf*Mig1^88^ strain using the ATMT procedure, and the hygromycin-resistant transformants (*Pf*OAO7) obtained were analyzed for gene integration and protein expression (Fig. 4b). PCR was performed to confirm the integration of the Egl16 cassette into the genome as shown in Fig. 4c. PCR reactions performed with the primers PgpdA-F and TrpC-R, which were designed based on vector sequence, generated the anticipated amplification products (~ 5 kb fragment) in three out of the five transformants selected, but not in the control *Pf*Mig1^88^, which confirmed the integration of the Egl16 cassette into the genome of *Pf*Mig1^88^ strain (Fig. 4c).

For assessment of the expression level of the overexpressed Egl16 gene, cultures of NCIM1228, *Pf*Mig1^88^, and the three positive transformants of *Pf*OAO7 were grown in Mandel’s media containing 25 g/L Avicel and 15 g/L SCB as carbon sources for 60 h for mRNA isolation and RT-PCR. The transcript levels of all five strains under derepressing conditions were evaluated with tubulin as a control, and the relative fold change was normalized to the level in NCIM1228 (Fig. 4d). The results showed that the transcript level of Egl16 remained almost unchanged for *Pf*Mig1^88^ despite the deletion of the carbon catabolite repressor gene in its genome while there were about threefold increases in transcript levels for two of the *Pf*OAO7 strains. To evaluate the expression level of the endoglucanase gene in the *Pf*OAO7 transformants, the three transformants obtained were cultivated in a cellulase-inducing medium (CIM) for 5 days and the supernatants of the harvested cultures were used for enzyme assays on mixed-linked glucan substrates. The results showed an enhancement in lichenase and laminarase activities in two of the transformants when compared with the parent strain while one of the transformants (T3) showed almost the same activity as that of the parent strain (Fig. 4e). About 26% and 18% increase in laminarase and lichenase activities were found with two of the transformants (T1 and T4) over that of *Pf*Mig1^88^. The transformant T4 gave the maximum laminarase and lichenase activities of 9.5 U/ml and 65 U/ml, respectively, while *Pf*Mig1^88^ yielded 7.5 U/ml and 54.8 U/ml for laminarase and lichenase, respectively. Evaluation of the impact of overexpressing this gene on the total cellulase activity showed that there were no changes in the FPase activity of the secretomes in comparison with that of the parent strain (Fig. 4e). This result was not surprising though as the overexpressed enzyme is an accessory enzyme that may show little or no activity on a pure cellulosic substrate such as Avicel^17^.

To evaluate the corresponding impact of overexpressing this gene on the quality of *Pf*Mig1^88^ secretome towards SCB hydrolysis, a hydrolysis reaction was set-up with a 15% solid load of SCB and 2.5 FPU/g DBW at 50 °C for 72 h. Upon completion of the hydrolysis, the resulting hydrolysates were analysed accordingly and the results obtained showed a significant increase in the glucose content after 72 h for the *Pf*OAO7 transformants while there was no difference in the total xylose released from the secretomes of the transformants when compared with the native strain (Fig. 4f), The results showed that the concentration of glucose released from SCB increased to 29.6 g/L and 28.5 g/L for transformants T1 and T4, respectively when compared with the control that yielded 26.1 g/L glucose. The approximate 13% increase in glucose yield with the *Pf*OAO7 transformants led to 42.8% cellulose conversion in contrast to *Pf*Mig1^88^ which was 37.7% cellulose conversion at this minimum enzyme dosage. Overall, the enhancement in saccharification performance of the *Pf*OAO7 transformants resulted in 46% total holocellulose conversion (Fig. 4g) which was ~ 7% improvement over that of the parent strain (43%). These results, therefore, indicate that the introduction of an extra copy of the Egl16 gene into the genome of the *Pf*Mig1^88^ strain enabled increased production of its corresponding enzyme for better hydrolysis on complex heterogeneous substrates such as SCB.

### Double overexpression of *Pf*xyn5 and *Pf*Egl16 results in an enriched accessory enzyme system for better saccharification of SCB residues

**Figure 5:**
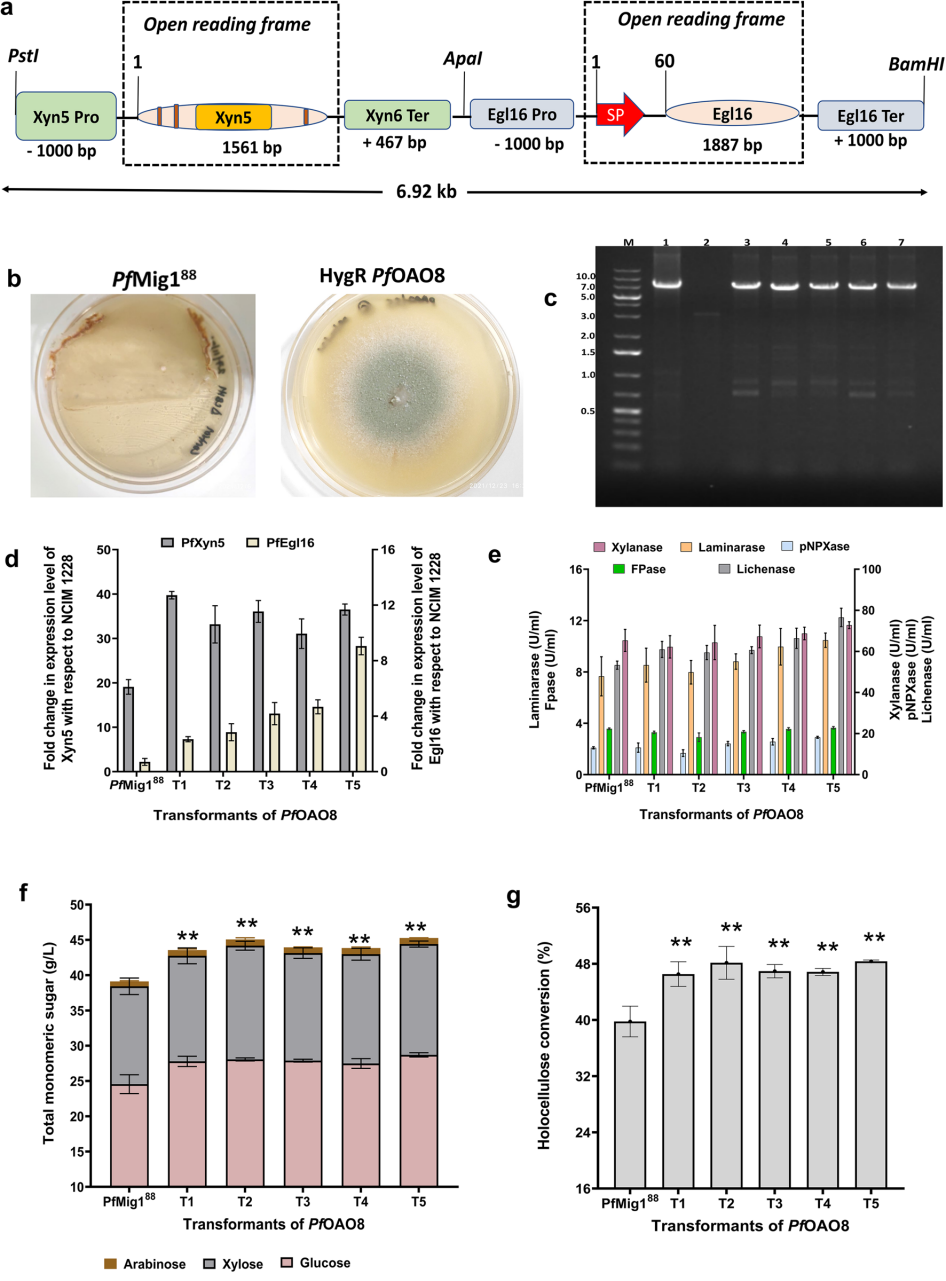
Vector construction for the simultaneous overexpression of *Pf*Xyn5 and *Pf*Egl16 in *Pf*Mig1^88^. (**a**) Schematic diagram showing the construction cassette for the dual overexpression of the Xyn5 and Egl16 genes from *P. funiculosum* NCIM1228. Abbreviations: Pro, promoter; SP, signal peptide; Ter, terminator. (**b**) Transformants of pOAO10 after AMTM transformation in *Pf*Mig1^88^. Transformants were selected on 100 µg/ml hygromycin. (**c**) Determination of integration of the dual cassette (Xyn5/Egl16) in the genome of *Pf*Mig1^88^ transformants by PCR using the primers PgpdA-F and TrpC-R. The expected size is 7 kbp, corresponding to a fragment spanning the region of gpdA promoter and TrpC on the expression cassette. Lane M is DNA molecular mass marker; lane (1) is the pOAO10 plasmid which served as a positive control while Lane (2) is the gDNA of *Pf*Mig1^88^ which was the negative control. Lanes 3–7 are the transformants of *Pf*OAO8. (**d**) The transcriptional expression of endoglucanase in NCIM1228, *Pf*Mig1^88^, and all the five positive strains of *Pf*OAO8 was measured by quantitative real-time PCR after growing the strains for 60 h in the presence of Avicel and SCB. The expression levels were normalized to those in NCIM1228 and plotted. (**e**) The xylanase, β-xylosidase, lichenase, laminarase and FPase activities in the fermentation broth of *Pf*Mig1^88^ and the five transformants of P*f*OAO8. (**f**) Saccharification performance of *Pf*OAO8 secretomes on SCB. Total sugar released from hydrolysis of SCB by secretomes of *Pf*Mig1^88^ and three transformants of *Pf*OAO8. (**g**) Percentage holocellulose conversion measured at 72 h saccharification period. ** shows the significant difference at α = 0.05 using Tukey’s multiple comparison test.

In the above sections, we found that individual overexpression of *Pf*xyn5 and *Pf*Egl16 provided remarkable hydrolysis efficiency against SCB at low enzyme dosage. The results provided an insight that simultaneously overexpressing the two genes may further facilitate enhanced deconstruction of SCB over what was achieved when individually overexpressed. To engineer the fungal strain for the dual overexpression of both Xyn5 and Egl16, a systematic approach was utilized for the construction of the desired *p*OAO10 vector containing the Xyn5/Egl16 expression cassette (Fig. 5a), as described in “Materials and methods”. For fungal transformation, the pOAO10 plasmid was confirmed by restriction digestion before transformation into the *Pf*Mig1^88^ strain (Supplementary Fig. S5c). The hygromycin-resistant transformants (*Pf*OAO8) obtained (Fig. 5b) were analysed by PCR with the primers PgpdA-F and TrpC-R which were designed based on vector sequence. The results showed that all the transformants generated the anticipated amplification products (~ 7 kb fragment) which was absent in the native strain thereby confirming the integration of the Xyn5/Egl16 cassette into the genome of the fungus (Fig. 5c).

A further evaluation of the transcript level of the overexpressed genes (Xyn5 and Egl16) showed increased xylanase abundance with the *Pf*OAO8 transformants over that of *Pf*Mig1^88^ when grown in Mandel’s media containing 25 g/L Avicel and 15 g/L SCB as carbon sources (Fig. 5d). Transformants T1 showed the highest increased Xyn5 transcript which was twofold higher than that of *Pf*Mig1^88^ while transformants T3 and T5 both showed a 1.8-fold increment in Xyn5 transcripts (Fig. 5d). For Egl16, the results obtained showed about three to ninefold increases in its transcript level after 60 h with transformants T5 having the highest Egl16 level (Fig. 5d).

To evaluate the expression level of the two overexpressed genes at the translational level, the positive transformants of *Pf*OAO8 were cultivated in a cellulase-inducing medium (CIM) for 5 days and the supernatants of the harvested cultures were used for enzyme assays as stated in “Materials and methods”. Analysis of the results showed significant improvement in enzyme activities across all the transformants when compared with the parent strain which was also in accordance with the mRNA levels (Fig. 5e). The *Pf*OAO8 transformants showed ~ 17% and ~ 7% increase in xylanase and β-xylosidase activities, respectively, compared to *Pf*Mig1^88^. Similarly, we found about 43% and 37% increases in lichenase and laminarase activities, respectively when compared to *Pf*Mig1^88^ while the FPase activities in all the transformants remained unchanged just as it was observed for the *Pf*OAO6 and *Pf*OAO7 strains.

For the evaluation of the corresponding impact of simultaneously overexpressing these two genes on the quality of *Pf*Mig1^88^ secretome towards SCB hydrolysis, another set of hydrolysis reactions was set up with a 15% solid load of SCB and 2.5 FPU/g DBW at 50 °C for 72 h. Upon completion of the hydrolysis, the resulting hydrolysates were analysed accordingly and the results obtained showed a significant increase in both the glucose and xylose contents of the hydrolysates for the *Pf*OAO8 transformants when compared with the native strain (Fig. 5f). The results showed between 11 and 17% increase in the concentration of glucose released for the *Pf*OAO8 transformants when compared with the control. Furthermore, the concentration of xylose in the hydrolysates of the *Pf*OAO8 transformants increased by 11%. The saccharification efficiency (holocellulose conversion) for *Pf*OAO8 strains when analysed was found to be considerably higher than the individual overexpressing strains (*Pf*OAO6 and *Pf*OAO7) as they exhibited between 45 and 49% holocellulose conversion in contrast to the native strain (*Pf*Mig1^88^) where ~ 40% holocellulose conversion was achieved (Fig. 5g). These results demonstrated that the Xyn5–Egl16 double overexpression provided a highly significant increase in SCB saccharification efficiency when compared to the individual ones and the engineered strains developed could potentially be used as promising bioresources needed for the production of more balanced cellulase and accessory enzyme cocktails required for the low-cost production of lignocellulose-based biofuels^34,36^.

In conclusion, an unknown glycosyl hydrolase 5 xylanase from the hypercellulolytic *P. funiculosum* was recombinantly expressed and characterized and its mechanism of action in the deconstruction of lignocellulose especially SCB was further deciphered in this study. The notable biochemical properties of *Pf*Xyn5 suggest it is a suitable candidate for various industrial applications most especially in bioenergy. Using a combined application of various fungal genetic tools, a new fungal strain was also created for simultaneous overexpression of notable accessory enzymes whose secretome demonstrated enhanced saccharification performance on pretreated SCB with minimal protein loading.

## Materials and methods

### Plasmids and microbial strains

All the strains and plasmids used in this study are listed in Table 4, while all the primer sets used in the study are itemized in Table 5. The nucleotide and protein sequences for Xyn5 and Egl16 for the study have been deposited in the NCBI database with accession numbers OM160816 and OM483862, respectively. *Escherichia coli* DH5α was used for plasmid propagation throughout the experiments. The *Agrobacterium tumefaciens* LBA4404 strain used for fungal transformation was maintained on a low-sodium LB medium (10 g/L tryptone, 5 g/L yeast extract, 5 g/L sodium chloride) containing 100 µg/ml kanamycin and 30 µg/ml rifampicin. The pBIF vector which was used as the backbone vector for fungal transformation, contains hygromycin and kanamycin resistance genes as selective markers for the selection of transformants (35). The pJET vector containing the ampicillin resistance gene was used as a shuttle vector for the sub-cloning of the Egl16 gene before re-cloning into pBIF for overexpression. *P. funiculosum* NCIM1228 and its derivative *P. funiculosum* Mig1^88^ (*Pf*Mig1^88^), the fungal strains used for this study, were routinely cultivated on Petri dishes containing low-malt extract–peptone (LMP) agar for approximately 14 days until full sporulation. For the selection and maintenance of fungal transformants, an LMP medium supplemented with hygromycin B at 100 µg/ml was used.

**Table 4:** List of plasmids and strains used in the study.

Strains	Description	References/sources
*Escherichia coli* DH5α	F-Φ80lacZΔM15 Δ(lacZYA-argF) U169 recA1 endA1 hsdR17	Invitrogen
*Agrobacterium tumefaciens* LBA4404	Ach5 with pAch5, Δ*tra* Δ*occ* ΔT_L_ ΔT_R_ Rif^r^	^33^
*Pichia pastoris* X-33	Wild type	Invitrogen^18^
*Penicillium funiculosum* NCIM1228	Cellulase-producing fungus obtained from NCIM (National Culture Collection Centre, Pune, India)	^18^
*Pf*Mig1^88^	Catabolite derepressed strain of *Penicillium funiculosum* NCIM1228	^33^
*Pf*OAO6	Xylanase (Xyn5) overexpression in *Pf*Mig1^88^ with the help of pOAO7 vector	This study
*Pf*OAO7	Endo-β-1,3(4) glucanase (Egl16) overexpression in *Pf*Mig1^88^ with the help of pOAO9 vector	This study
*Pf*OAO8	Xylanase and endo-β-1,3(4) glucanase co-overexpression in *Pf*Mig1^88^ with the help of pOAO10 vector	This study
**Plasmids**		
*p*PICZαA	A zeocin-resistant vector for protein expression in *P. pastoris* under the control of AOX1 promoter	Genscript
*p*OAO6	A pPICZ_α_A vector containing the *Pf*GH5 gene under the control of AOX1 promoter	This study
*p*BIF	Kanamycin and hygromycin resistant vector containing EGFP	^37^
*p*JET	Standard cloning vector containing the ampicillin-resistant gene	XB-VEC-1221267
*p*OAO7	pBIF vector containing the *Pf*Xyn5 gene along with its native promoter and terminator	This study
*p*OAO8	pJET vector containing the endo-β-1,3(4) glucanase gene along with its native promoter and terminator	This study
*p*OAO9	pBIF vector containing endo-β-1,3(4) glucanase gene along with its native promoter and terminator	This study
*p*OAO10	pBIF vector containing xylanase and endo-β-1,3(4) glucanase genes along with their native promoter and terminator	This study

**Table 5:** List of primers used in the study.

Screening of *Pf*GH5 transformants in *Pichia pastoris*	AOX1-F (5′-GACTGGTTCCAATTGACAAGC-3′)
	AOX1-R (5′-GCAAATGGCATTCTGACATCC-3′)
*Pf*Xyn5 and *Pf*Egl16 plasmid construction cassette	PfXyn5-F (5′-TCCAGACTGCAGAACAGAGATCTCACGGTC-3′)
	PfXyn5-R (5′-TTCCAGGATCCTTCTCTTCCGGTCCCAG-3′)
	PfEgl16-F1 (5′-TCAGTGAGCTCAGTTGGCAGAAGACGAAGCAG-3′)
	PfEgl16-R1 (5′-TATACACGCGTGGCGACGAATTCCGCGAC-3′)
	PfEgl16-F2 (5′-TCAGTGGGCCCAGTTGGCAGAAGACGAAGCAG-3′)
	PfEgl16-R2 (5′-TACTGGATCCGGCGACGAATTCCGCGAC-3′)
	PgpdA-F (5′-GAATTCTGTACAGTGACCGGTGACTC-3′)
	TrpC-R (5′-GCCAAGCTTCCTCTAAACAAGTGTACCTGTGC-3′)
	PgpdA IR-F (5′-TGCGTCAGTCCAACATTTGT-3′)
RT-PCR primers	PfXyn5 RT F (5′-CTGGATCAGATTCCGTCGAAATTTTATTCGG-3′)
	PfXyn5 RT R (5′-CCATCGGCTCCCCACAGATC-3′)
	PfEgl16 RT F (5′-CTACAAGCAGCCCTAGTACTGTGACAG-3′)
	PfEg16 RT R (5′-GTAAAGACGGGAGCAATGCTGCT-3′)
	Tubulin F (5′-ATTGCTCAGGTTGTCTCCTCCATC-3′)
	Tubulin R (5′-CATGGTGATCTCGTTGACAGAGTTGG-3′)

The underlines are the site of the restriction enzyme used for cloning.

### Cellulosic substrate and pretreatment

NaOH-treated Sugarcane bagasse was used as the cellulosic substrate in this study. The SCB was kindly provided by Natems Sugars Private Limited, Hyderabad, Telangana, India. SCB was washed, dried in a convection oven at 60 °C to constant weight, and then knife-milled using a 1 mm sieve in the mill. The alkaline pretreatment of SCB was done at 90 °C using 0.4 M NaOH at 10% solid loading for 6 h. After pretreatment, the solids were washed three times was then repeatedly washed until the pH became neutral. Compositional analysis conducted according to National Renewable Energy Laboratory (NREL) procedure TP510-42618^38^ on the pretreated sugarcane bagasse yielded a cellulose content of 46.1%, a hemicellulose content of 24.5% and a lignin content of 17%.

### Computational analysis of Xyn5 from *P. funiculosum* (*Pf*Xyn5)

The Xyn5 sequence was retrieved from the draft genome sequence of *P. funiculosum* NCIM1228 available in our laboratory. The amino acid sequences of some closely similar glycoside hydrolase 5 proteins from other species were retrieved from the NCBI database, and molecular phylogenetic analysis by the maximum likelihood method and the Tamura-Nei model was conducted using MEGA X software^39^. Multiple-sequence alignment of *Pf*Xyn5 with other GH5 proteins obtained from the phylogenetic analysis was performed using the Clustal Omega multiple-sequence alignment program^40^. The signal peptide and conserved domains were analyzed at Signal P 5.0 server (http://www.cbs. dtu.dk/services/SignalP/) and ScanProsite (http://www.expasy.ch/tools/ScanProsite), respectively. N-Glycosylation and O-glycosylation sites were predicted using NetNGlyc 1.0 (http://www.cbs.dtu.dk/services/NetNGlyc/) and NetOGlyc 4.0 server (http://www.cbs.dtu.dk/services/NetOGlyc/), respectively.

### Construction of *Pf*Xyn5 expression vector for recombinant expression and purification in *P. pastoris*

The xylanase sequence used in the present study from *P. funiculosum* was synthesized by GenScript USA, Inc. (New Jersey, USA) with codon optimization for expression in *P. pastoris*. The synthesized sequence was ligated into the vector *p*PICZαA, named *p*PICZαA-PfGH5 (or *p*OAO6). The *p*OAO6 vector was transformed into *Escherichia coli* (DH5α), and subsequently, the positive clone was screened, and the plasmid was extracted using Qiagen mini-prep kit. The *p*OAO6 plasmid was linearized using *Sac*I restriction endonuclease and transformed into *P. pastoris* X-33 competent cells using the lithium chloride method described by Kumar et al.^41^. The transformation liquid was plated on a YPD plate containing 100 µg/ml zeocin, and the positive transformants were further reselected on a higher concentration of zeocin (1000 µg/ml) for 48 h before being screened using polymerase chain reaction (PCR) with the AOXI-F and AOX1-R primer pairs (Table 5). The selected positive transformants were subsequently inoculated into buffered glycerol-complex medium (BMGY) for 24 h before transferring into methanol-complex medium (BMMY) for xylanase expression with the addition of 1% methanol every 12 h till the end of the fermentation process. The induction of the expressed protein was monitored by collecting samples from the cultures every 24 h and loaded on sodium dodecyl sulfate–polyacrylamide gel electrophoresis (SDS-PAGE).

To purify the protein, the culture supernatants were recovered by pelleting the cells by centrifugation at 4000 rpm for 5 min, 4 °C and filtered on 0.45-µm filters (Millipore, Molsheim, France) to remove any remaining cells. The recovered supernatant was dialyzed against 50 mM sodium phosphate buffer, pH 7.5 overnight with two equal changes of buffer at 12 h intervals until the pH becomes 7.5. After adjusting the pH to 7.5, the supernatants were filtered once more on 0.2-µm filters and loaded onto a FPLC HiTrap Q Sepharose XL 5 ml column (GE Healthcare) that was equilibrated with 50 mM sodium phosphate buffer (pH 7.5). Proteins were eluted using a linear gradient of NaCl (25–1000 mM) in the same buffer mentioned above at flow rate of 2.0 ml/min. Fractions containing the enzyme activity were pooled and subjected to SDS-PAGE to check protein purity. Protein concentrations were determined using the bicinchoninic acid (BCA) method using bovine serum albumin (BSA) as standard^14^.

### Biochemical characterization of recombinant *Pf*Xyn5

To characterize the recombinant *Pf*Xyn5, the protein was screened on various polysaccharides which include Avicel, beechwood xylan, xyloglucan, laminarin, sodium carboxymethyl cellulose, lichenan, β-glucan, p-nitrophenyl-β-d-glucopyranoside (pNPG) and p-nitrophenyl-β-d-xylopyranoside (pNPX). The substrates were incubated with the appropriate amount of the enzyme at 50 °C for 30 min and the amount of reducing sugar liberated was quantitated using the DNS method where appropriate. For determination of the mechanism of action of the enzyme, hydrolysis reactions were set up using 5% xylan and 5% pretreated SCB as substrates and protein dosage of 25 mg/g DBW at 50 °C for 24 h and 72 h, respectively. The resulting hydrolysates obtained were analysed using an Agilent 1260 series HPLC instrument coupled with a 1290 Infinity evaporative light scattering detector (ELSD). The degradation products were estimated using a Rezex RSO-oligosaccharide Ag^+^ (4%) column (200 by 10 mm) with a Rezex RSO-oligosaccharide Ag^+^ (4%) guard column (60 by 10 mm) (Phenomenex), with a mobile phase of 100% water. Throughout the analysis, the column was kept at 80 °C, and the mobile phase flow rate was maintained at 0.3 ml/min under isocratic conditions for 60 min. The chromatographic run was initiated by first equilibrating the column for 5 min followed by injecting 20 µl of the sample solution into the column. The ELSD was maintained at 45 °C throughout. The nebulizer (nitrogen) gas pressure was set at 2.8 × 10^5^ Pa, and the detector gain was set at 9 × 10^5^ Pa. The concentration of different XOS generated was calculated from calibration curves of external standards purchased from Megazyme. The pH dependence of *Pf*Xyn5 was determined by using 50 mM citrate–phosphate buffer (pH 3.0–6.0) containing purified enzyme and xylan at 50 °C as described above. To determine the effect of pH on the stability of *Pf*Xyn5, the enzyme was incubated in relevant buffers of varying pH (3.0–6.0) without substrate for 24 h at room temperature. The residual xylanase activity was determined according to the standard assay procedure earlier described. The temperature dependence was determined by incubating the reaction with the optimal pH at 40–80 °C. To determine the dependence on pH and temperature, the maximum activity obtained was defined as 100%. The thermal stability of *Pf*Xyn5 was analysed by preincubating it at 40–80 °C for 5 h and its residual activity was then determined.

### Effect of *Pf*Xyn5 xylanase supplementation on the performance of secretome from the *P. funiculosum *(*Pf*Mig1^88^) during biomass hydrolysis

The effect of supplementing the cellulase cocktail present in the secretome of *P. funiculosum* with *Pf*Xyn5 in SCB saccharification was determined by setting up hydrolysis reactions with pretreated 5% solid loading and a total protein dosage of 10 mg/g DBW at 50 °C for 72 h with constant shaking. The 10 mg/g protein concentration was maintained at different combination ratios of 60:40, 70:30, 80:20, 90:10, 95:05 and 100:0 for *P. funiculosum* to *Pf*Xyn5. After hydrolysis the samples were centrifuged to separate the solid residue from the digested biomass. Supernatants that were recovered after enzymatic hydrolysis of the pretreated SCB were analysed by high-performance liquid chromatography with an Aminex HPX-87H anion-exchange column (Bio-Rad, USA) and a refractive index (RI) detector to analyse the released monosaccharides (glucose and xylose) by anion-exchange chromatography. The concentration of each monosaccharide was calculated from calibration curves of external standards (xylose and glucose) purchased from Absolute Standards Inc. The degree of synergism between the *Pf*Xyn5 and cellulase was calculated following the method described by Goncalves et al. and Li et al.^13,32^ with the formula:

$${\text{Degree of synergism }}\left( {{\text{DS}}} \right) = \frac{{{\text{Y}}_{{{1} + {2}}} }}{{\left( {\upalpha {\text{Y}}_{{1}} + \upbeta {\text{Y}}_{{2}} } \right)}}$$

where α and β correspond to the mass ratio of the enzymes in each reaction. Y_1__+2_, indicates the yield of reducing sugar achieved by the two enzymes working simultaneously, and Y_1_ and Y_2_ indicate the yields of reducing sugar, achieved by each enzyme working separately.

### Engineering of ***P. funiculosum*** Mig1^88^ for overexpression of Xyn5 and Egl16

All vectors for fungal expression described in this study were constructed on the backbone of the *p*BIF vector that was previously constructed using the binary vector pCAMBIA1300 as a backbone^37^. Binary vectors for the overexpression of the Xyn5 and Egl16 genes from *P. funiculosum* were constructed as follows. The endogenous gene encoding Xyn5 was amplified from the genome of *P. funiculosum* NCIM1228 using the primers PfXyn5-F and PfXyn5-R (Table 5). The primers were designed according to the Xyn5 sequence containing both its native promoter and terminator, which was obtained from the draft genome sequence of the strain. The PCR product obtained was digested with the restriction enzymes PstI and BamHI before being ligated into the pBIF vector previously digested with the same enzyme sets to generate the *p*BIF-Xyn5 (*p*OAO7) vector. For the overexpression of Egl16, the Egl16 gene was amplified from the genome of *P. funiculosum* NCIM1228 using the primers PfEgl16-F1 and PfEgl16-R1 spanning its native promoter and terminator (Table 5). The PCR product was first subcloned into *p*JET1.2 to obtain the *p*OAO8 vector. The *Pst*I/*Xba*I fragment containing the Egl16 fragment was excised and cloned into the same sites in *p*BIF to create the *p*BIF-Egl16 vector (*p*OAO9) vector. The ligated products of *p*OAO7 and *p*OAO9 were then transformed into *E. coli* DH5α cells and selected on 50 µg/ml kanamycin. The resulting colonies were screened for positive transformants by colony PCR, followed by restriction digestion of the corresponding plasmids. To create a strain for the combined overexpression of both the Xyn5 and Egl16 genes, the *p*OAO7 vector was used as the base vector upon which the Egl16 gene was cloned. The Egl16 gene with its native promoter and terminator was re-amplified from the genome of *P. funiculosum* NCIM1228 using the primers PfEgl16-F2 and PfEgl16-R2 containing the *Apa*I and *Bam*HI restriction sites, respectively. The PCR product obtained was digested with the *Apa*I and *Bam*HI restriction enzymes before being ligated into the corresponding sites of the *p*OAO7 vector to obtain the pBIF-Xyn5/Egl16 (pOAO10) vector. The verified *p*OAO7, *p*OAO9, and *p*OAO10 plasmids were transformed into the *Pf*Mig1^88^ fungal strain according to the agrobacterium-mediated transformation method (AMTM) as previously described^42^. After transformation, the resulting hygromycin-resistant transformants were screened according to the method described previously by Fang and Xia^43^. The transformants were verified by PCR for the integration of the Xyn5 and Egl16 gene expression cassettes. For PCR analysis, the primers PgpdA-F and TrpC-R were used to screen for Xyn5 and Egl16 integration, while transformants of Xyn5/Egl16 were screened using the primer set PgpdA IR-F and TrpC-R (Table 5).

### Expression analysis of cellulolytic enzymes via real-time PCR

For real-time PCR experiments, cultures of all strains were grown in minimal Mandel’s medium containing 25 g/L Avicel and 15 g/L SCB for 60 h^44^. Mycelia were harvested by filtration and frozen in liquid nitrogen. RNA was extracted using an RNeasy kit (Qiagen) according to the manufacturer’s instructions. RNA was treated with DNase (Invitrogen) before cDNA synthesis. One microgram of RNA was used as the template for each quantitative real-time PCR (qRT-PCR). A cDNA synthesis control was performed to ensure the absence of DNA contamination. qRT-PCR was carried out using iTaq universal SYBR green supermix (Bio-Rad) and a Bio-Rad CFX96 qPCR detection system. Primers for transcripts to be tested were designed using the boundary sequence of two exons to avoid any amplification from contaminant genomic DNA. qRT-PCR was performed in biological triplicates with tubulin as the endogenous control. Relative expression levels were normalized to the level of tubulin, and the fold changes in RNA levels were calculated as the ratios of the relative expression levels in *Pf*Mig1^88^ and the corresponding transformants of Xyn5 and Egl16 to that in NCIM1228 under cellulase-inducing conditions^45^.

### Cellulolytic secretome preparation

*Penicillium funiculosum* NCIM1228, *Pf*Mig1^88^, and the resulting *Pf*OAO6, *Pf*OAO7, and *Pf*OAO8 transformants were cultivated on Petri dishes containing low-malt extract agar until full sporulation. After 14 days of incubation, spores were recovered with sterile water, filtered through sterile Mira cloth, and quantified using a hemocytometer. The primary culture of each strain was prepared by culturing 10^7^ conidiophores in potato dextrose broth (PDB) for 36 h. Primary cultures of the strains were added to a cellulase-inducing medium in Erlenmeyer flasks at a final concentration of 10%. Cellulase-inducing medium (CIM) contained soya peptone (24 g/L), wheat bran (14 g/L), microcrystalline cellulose (MCC) (16 g/L), pretreated SCB (15 g/L), KH_2_PO_4_ (12.4 g/L), K_2_HPO_4_ (2.68 g/L), (NH_4_)_2_SO_4_ (0.28 g/L), CaCO_3_ (2.5 g/L), corn steep liquor (1%), urea (0.52 g/L), and yeast extract (0.05 g/L), with the final pH adjusted to 5.0. The flasks were kept at 28 °C for 5 days with orbital shaking at 150 rpm (Innova 44; Eppendorf AG, Germany). Induced cultures were centrifuged at 9000 rpm for 10 min at 4 °C, and the cellulolytic supernatants were collected and stored at 4 °C until use.

### Determination of the enzyme activities of the engineered secretome

All enzymatic activities performed in this study were routinely determined following standard assay procedures. Endoglucanase, xylanase, lichenase, laminarase, β-glucosidase and β-xylosidase activities were determined by incubating appropriate dilution of the enzyme with 2% CMC (Sigma), 2% beechwood xylan (HiMedia), 1% lichenan (Megazyme), 1% laminarin (Sigma) and p-nitrophenyl-β-d-xylopyranoside (Megazyme), respectively, for 30 min, after which the amount of reducing sugars released was measured as previously reported^12^. One unit of CMCase, xylanase, lichenase and laminarase activity is defined as the amount of enzyme releasing 1 µmol of reducing sugar per min while one unit of β-xylosidase activities was defined as the amount of protein that released 1 μmol of p-nitrophenol (pNP) per min. Total cellulase activity in the secretome was measured in terms of filter paper units (FPU) per milliliter of original (undiluted) enzyme solution. The assay requires a fixed degree of conversion of the substrate, from 50 mg of filter paper within 60 min at 50 °C. One FPU is defined as the amount of enzyme required to produce 2 mg of glucose from 50 mg of filter paper within 60 min of incubation^46^. To quantitate the amount of secreted proteins in the various *Pf*Mig1^88^ secretomes as well as its derivatives, the crude secretomes were first buffer exchanged with the help of a 10-kDa cut-off membrane using citrate– phosphate buffer (pH 4.0) and then the total protein of each secretome was estimated by the bicinchoninic acid (BCA) method using bovine serum albumin (BSA) as standard.

### Biomass saccharification and quantification of fermentable sugars

The saccharification efficiency of the secretomes of all the strains used in the study with pretreated SCB was determined according to the method described previously by Ogunyewo et al.^34^, with some modifications. The performance of the secretomes toward NaOH-treated SCB was evaluated at 15% dry weight of biomass using an enzyme concentration of 2.5 FPU/g DBW. Saccharification was performed in 50 ml screw-cap Falcon tubes in an incubator shaker at 50 °C for 72 h. The reaction mixture included NaOH-treated SCB under 15% dry weight loading in a 5 ml final reaction volume. The total cellulase activities of each secretome of all the fungal strains tested were measured, and the appropriate volume of the desired protein concentration (2.5 FPU/g DBW) was added to the reaction mixture. The reactions were set up in 50 mM citrate phosphate buffer (pH 4.0), and the mixtures were incubated at 50 °C with constant shaking at 220 rpm for 72 h. Samples were collected at the end of the 72 h incubation period and analysed for the production of fermentable sugars. Control experiments were carried out under the same conditions, using substrates without enzymes (enzyme blank) and enzymes without substrates (substrate blank); a substrate-free negative control was set up by filling the Falcon tubes with 50 mM citrate phosphate buffer (pH 4.0), and the background of soluble sugars present in the respective biomass was determined by incubating each biomass in the absence of the enzyme. Following the completion of hydrolysis at each time point, the Falcon tubes were centrifuged at 3500 rpm for 10 min in a swinging-bucket centrifuge (Eppendorf, Germany) to separate the solid residue from the digested biomass. Supernatants that were recovered after enzymatic hydrolysis of the pretreated SCB were analysed by high-performance liquid chromatography with an Aminex HPX-87H anion-exchange column (Bio-Rad, USA) and a refractive index (RI) detector to analysed the released monosaccharides (glucose and xylose) by anion-exchange chromatography. The filtered mobile phase (4 mM H_2_SO_4_) was used at a constant rate of 0.3 ml/min with the column, and the RI detector temperature was maintained at 35 °C. The concentration of each monosaccharide was calculated from calibration curves of external standards (xylose and glucose) purchased from Absolute Standards Inc. The theoretical conversions of cellulose and hemicellulose (in percentage) into monomeric sugars were calculated using the equations provided in NREL’s LAP TP-510-43630 as earlier reported^19^.

### Data and statistical analysis

All experiments were performed in triplicate, and the results are presented as the means and standard deviations. The data were compiled in a Microsoft Excel spreadsheet, where the averages and standard errors of the means were determined. All graphs were created using GraphPad Prism 8.0 software. The data were further evaluated by one-way analysis of variance (ANOVA) and multiple t-tests using GraphPad Prism 8.0 software where appropriate.

## Supplementary Information

Supplementary Figures.

## Data Availability

The nucleotide and protein sequences for the *Pf*Xyn5 and *Pf*Egl16 genes used for this study have been deposited to the NCBI database with accession numbers OM160816 and OM483862, respectively.
