# Supplementary material for: Characterization of a GH5 endoxylanase from Penicillium funiculosum and its synergism with GH16 endo-1,3(4)-glucanase in saccharification of sugarcane bagasse
Source: Sci Rep. 2022 Oct 14;12:17219. doi: 10.1038/s41598-022-21529-1 (PMC9568505; doi:10.1038/s41598-022-21529-1)
Supplement: Supplementary file 1 — Supplementary Figures. [file 41598_2022_21529_MOESM1_ESM.pdf]

Supplementary Information

for

Manuscript entitled "Characterization of a novel GH5 endoxylanase from *Penicillium funiculosum* and its synergism with GH16 endo-1,3(4)-glucanase in saccharification of sugarcane bagasse"

Olusola A. Ogunyewo, Omoaruemike E. Okereke, Sandeep Kumar and Syed Shams Yazdani

List of Items:

Figure S1: Molecular screening of GH5 transformants after transformation in *Pichia pastoris* X-33

Figure S2: Expression and Purification of recombinant *Pf*Xyn5 in *P. pastoris*

Figure S3: Analysis of the degradation products of pretreated sugarcane bagasse with recombinant PfXyn5

Figure S4: Half-life of PfGH5 after incubation at different temperatures

Figure S5: Molecular confirmation of recombinant vectors for fungal transformation

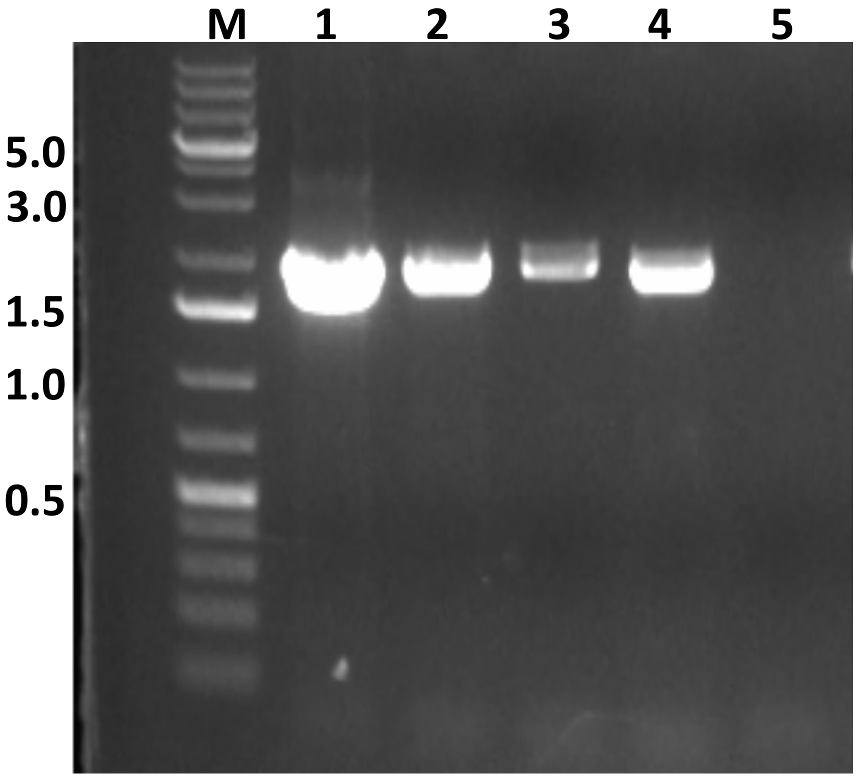

Figure S1: Molecular screening of GH5 transformants after transformation in *Pichia pastoris* X-33. Colonies were selected on 1000 µg/ml zeocin after 48 h. Lane 1 is the pOAO6 vector (positive control), Lanes 2 – 4 refers to the GH5 transformants while Lanes 5 is the wild type strain of *P. pastoris* X-33.

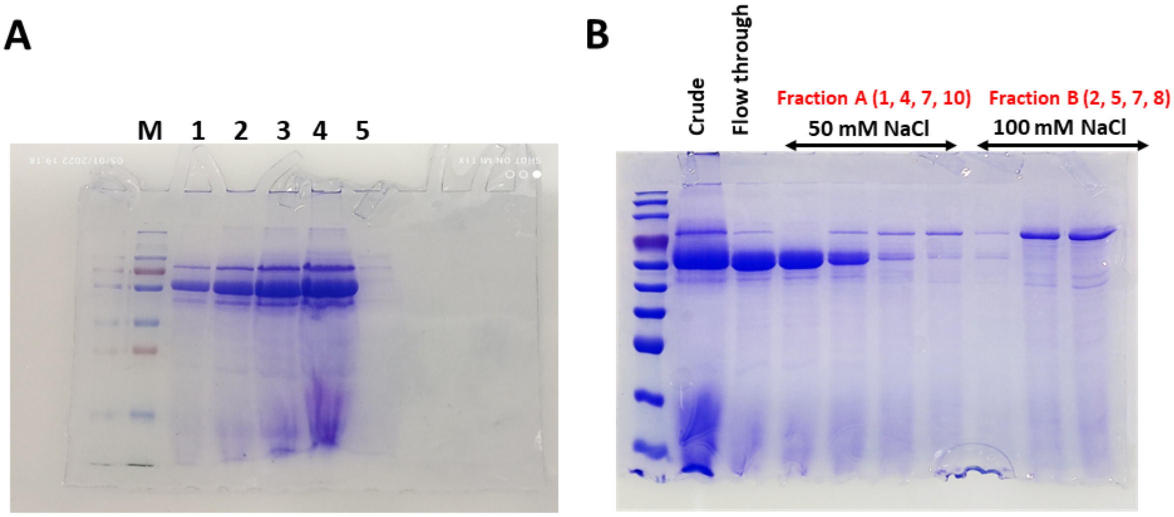

Figure S2: Expression and Purification of recombinant *Pf*Xyn5 in *P. pastoris* (a) SDS-PAGE gel of the supernatant of PfXyn5 produced by *P. pastoris* over 96 h period of induction. Lanes 1- 4 refers to induced cultures of PfXyn5 taken at 24, 48, 72 and 96 h time point while lane 5 refers to wild type culture of *P. pastoris* X-33 at 96 h time point. (b) SDS-PAGE gel of purified fractions of *Pf*Xyn5 by anion exchange chromatography using Q-Sepharose Fast Flow resin.

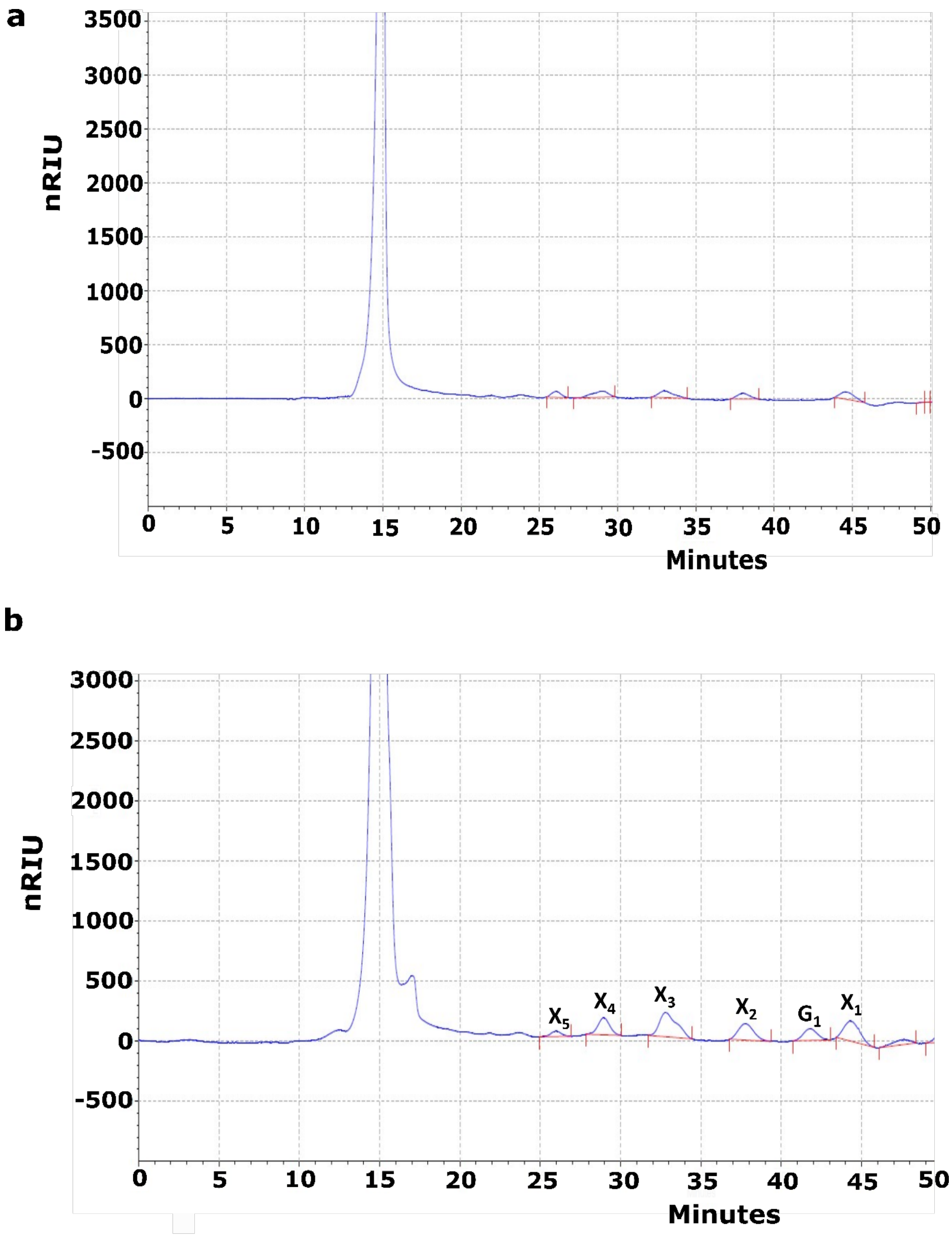

Figure S3: Analysis of the degradation products of pretreated sugarcane bagasse with recombinant *Pf*Xyn5 (a) HPLC profile for the hydrolysis reaction without *Pf*Xyn5 (control) (b) HPLC profile of hydrolyzed products of sugarcane bagasse with PfXyn5. X_1, X_2, X_3, X_4, X_5 and X_6 refers to xylose, xylobiose, xylotriose, xylotetrose, xylopentaose and xylohexaose, respectively while G_1 refers to glucose.

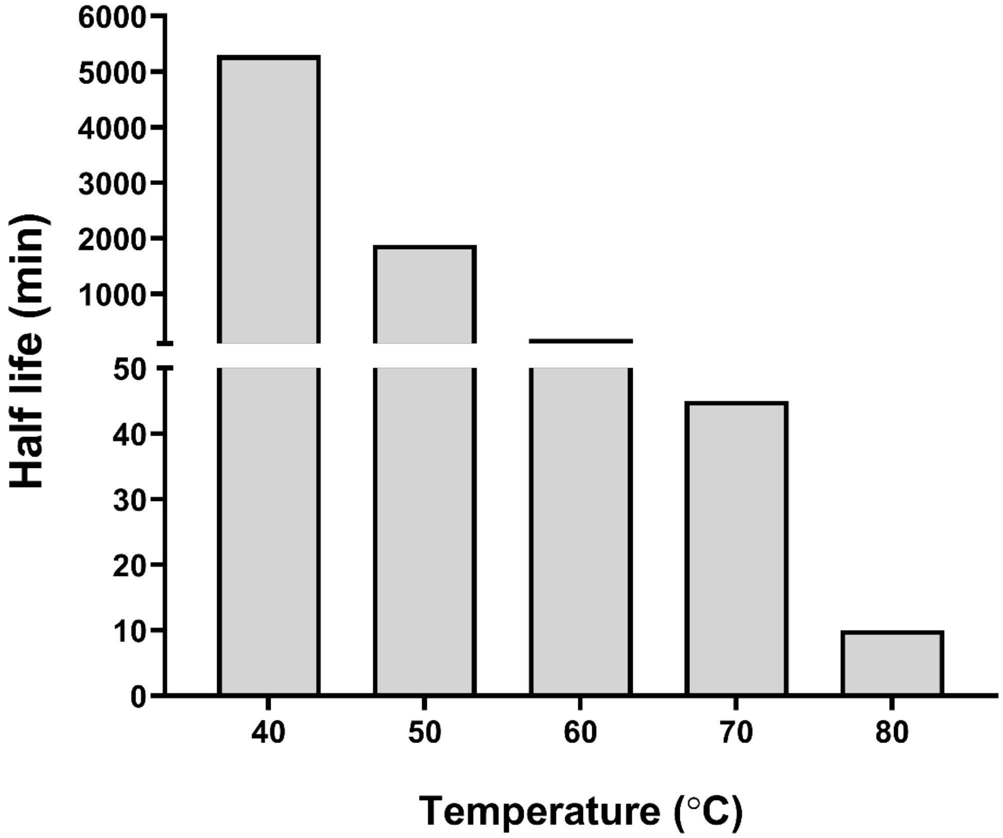

Figure S4: Half-life of PfGH5 after incubation at different temperatures

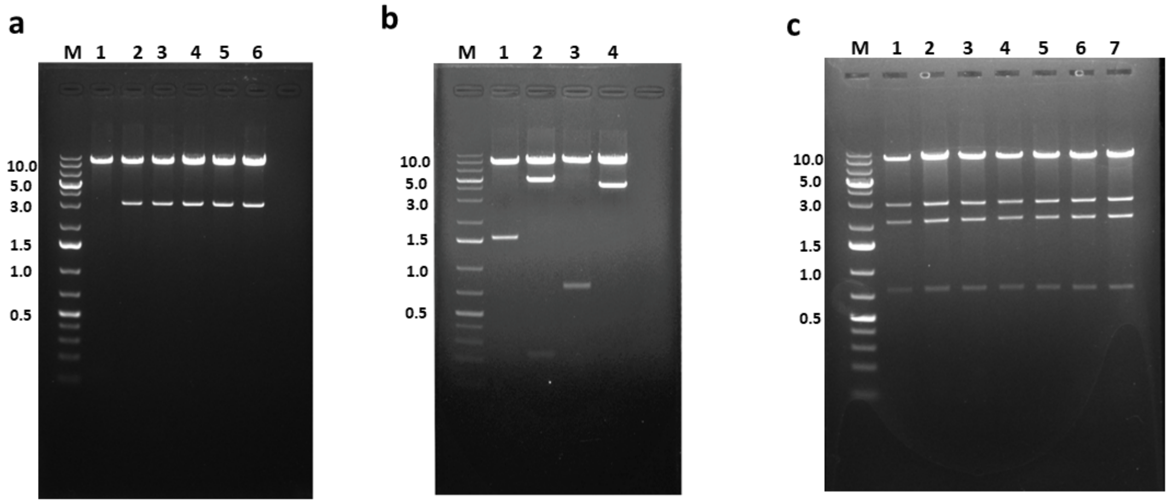

Figure S5: Molecular confirmation of recombinant vectors for fungal transformation. (a) restriction digestion analysis of *p*BIF (1) and *p*OAO7 (2-6) by *PstI and ApaI.* (b) restriction digestion analysis of *p*BIF (1) and *p*OAO9 (2) by *MunI and MluI; p*BIF (3) and *p*OAO9 (4) by Pst*I and XbaI* (c) restriction digestion analysis of *p*OAO10 (1-7) by *PstI and ApaI.*
